# *N*-Glycans: Phenotypic Homology and Structural Differences between Myocardial Cells and Induced Pluripotent Stem Cell-Derived Cardiomyocytes

**DOI:** 10.1371/journal.pone.0111064

**Published:** 2014-10-30

**Authors:** Takuji Kawamura, Shigeru Miyagawa, Satsuki Fukushima, Akira Yoshida, Noriyuki Kashiyama, Ai Kawamura, Emiko Ito, Atsuhiro Saito, Akira Maeda, Hiroshi Eguchi, Koichi Toda, Jong-Kook Lee, Shuji Miyagawa, Yoshiki Sawa

**Affiliations:** 1 Department of Cardiovascular Surgery, Osaka University Graduate School of Medicine, Suita, Osaka, Japan; 2 Department of Cardiovascular Regenerative Medicine, Osaka University Graduate School of Medicine, Suita, Osaka, Japan; 3 Division of Organ Transplantation, Department of Surgery, Osaka University Graduate School of Medicine, Suita, Osaka, Japan; Tokai University, Japan

## Abstract

Cell surface glycans vary widely, depending on cell properties. We hypothesized that glycan expression on induced pluripotent stem cells (iPSCs) might change during cardiomyogenic differentiation toward the myocardial phenotype. *N*-glycans were isolated from iPSCs, iPSC-derived cardiomyocytes (iPSC-CM), and original C57BL/6 mouse myocardium (Heart). Their structures were analyzed by a mapping technique based on HPLC elution times and MALDI-TOF/MS spectra. Sixty-eight different *N*-glycans were isolated; the structures of 60 of these *N*-glycans were identified. The quantity of high-mannose type (immature) *N*-glycans on the iPSCs decreased with cardiomyogenic differentiation, but did not reach the low levels observed in the heart. We observed a similar reduction in neutral *N*-glycans and an increase in fucosylated or sialyl *N*-glycans. Some structural differences were detected between iPSC-CM and Heart. No *N*-glycolyl neuraminic acid (NeuGc) structures were detected in iPSC-CM, whereas the heart contained numerous NeuGc structures, corresponding to the expression of cytidine monophosphate-*N*-acetylneuraminic acid hydroxylase. Furthermore, several glycans containing Galα1-6 Gal, rarely identified in the other cells, were detected in the iPSC-CM. The expression of *N*-glycan on murine iPSCs changed toward the myocardial phenotype during cardiomyogenic differentiation, leaving the structural differences of NeuGc content or Galα1-6 Gal structures. Further studies will be warranted to reveal the meaning of the difference of *N*-glycans between the iPSC-CM and the myocardium.

## Introduction

*In vitro* generation of cardiac myocytes by reprogramming is a promising technology in developing cell-transplant therapy for advanced cardiac failure [1] and drug discovery for a variety of cardiac diseases [2]. For both purposes, induced pluripotent stem cells (iPSCs) are most useful, since generation and cardiomyogenic differentiation of iPSCs has been standardized in human and a number of animals [3], [4]. In fact, derivatives of iPSCs have been developed to the pre-clinical stage for cell transplantation therapy [5], while cardiac myocytes generated from patient-specific iPSCs have been studied to explore pathologic mechanisms and guide drug discovery [6], [7]. However, cardiac myocyte preparations from iPSCs contain immature phenotypes, observed by electrophysiology, electron microscopy, and immunohistochemistry [8], [9]; this may limit the safety and efficacy of cell transplantation therapy or reduce the accuracy and efficiency of drug discovery. The maturity of iPSC-derived cardiac myocytes (iPSC-CMs) has not been comprehensively or quantitatively evaluated.

Cell surface glycans have several important functions interacting with numerous proteins, including growth factors, morphogens and adhesion molecules, modulating dynamic cellular mechanisms such as cell-cell adhesion, cell activation, and malignant alterations [10]–[12]. In early mammalian embryos, associated with fertilization, some *N*-glycans play important roles of cell-cell adhesion [13]–[15]. In addition, cellular responsiveness to growth or arrest depends on total *N*-glycan number and the degree of branching of cell surface glycoproteins [16]. Furthermore, heparan sulfate, a kind of glycans, is required for embryonic stem cell (ESC) pluripotency, in particular lineage specification into mesoderm through facilitation of FGF and BMP signaling by stabilizing BMP ligand [17], leading the evidence that the expression patterns of cell surface glycans on ESCs changes during differentiation [18]. Thus, we hypothesized that cell surface glycan expression may change during the course of cardiomyogenic differentiation of iPSCs *in vitro*. We analyzed *N*-glycan expression in undifferentiated iPSCs, iPSC-CMs, and adult murine myocardium by HPLC, to identify potential indicators of the maturity of differentiating cardiomyocytes from iPS cells *in vitro*.

## Materials and Methods

Animal care procedures were consistent with the “Guide for the Care and Use of Laboratory Animals” (National Institutes of Health publication). Experimental protocols were approved by the Ethics Review Committee for Animal Experimentation of Osaka University Graduate School of Medicine.

### Cardiomyogenic differentiation of murine iPSCs *in vitro*

We used the murine iPSC lines, 959A2-1, 959C1-1, 956F-1 (generous gifts from Dr. Okita and Professor Yamanaka of the Center for iPS Cell Research and Application, Kyoto University, Kyoto, Japan). The cell lines were generated from C57BL/6 (B6) (CLEA) mouse embryonic fibroblasts by introducing *Oct3/4, Sox2, Klf4*, and *c-Myc* without viral vectors as described [19]. The iPSCs were cultured in the absence of serum and feeder cells by using ESGRO Complete PLUS Clonal Grade Medium (Millipore).

**Figure 1:**
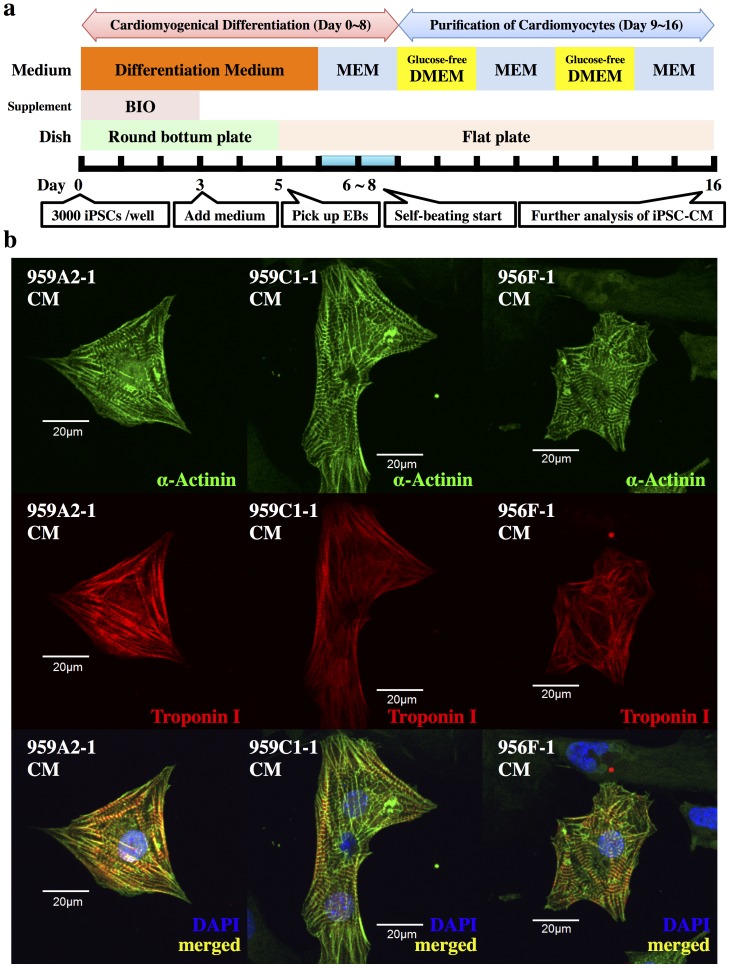
Cardiomyogenic differentiation of iPSCs and cardiomyocyte purification. (a) The cardiomyogenic differentiation protocol and cardiomyocyte purification process are illustrated. (b) IPSC-CMs stained with anti-α-actinin antibody (Alexa Fluor 488), anti-troponin I (Alexa Fluor 594) and DAPI, were analyzed with a confocal laser scanning microscopy. Abbreviations: EB, embryonic body; MEM, Modified Eagle's Medium; DMEM, Dulbecco's Modified Eagle's Medium; BIO, 6-bromoindirubin-3′-oxime.

Cardiomyogenic differentiation of the iPSCs was performed as described [20], [21], with modifications, followed by purification with glucose-free medium supplemented with lactic acid [22]; iPSCs (3 × 10^3^) were resuspended in 100-µL aliquots of differentiation medium [DM; Dulbecco's Modified Eagle's Medium (DMEM; Nacalai Tesque) containing 15% fetal bovine serum (FBS; Biofill), 100 µmol/L non-essential amino acids (NEAA; Invitrogen), 2 mmol/L L-glutamine (Invitrogen), and 0.1 mmol/L 2-mercaptoethanol (Invitrogen)] containing 0.2 µmol/L 6-bromoindirubin-3′-oxime (BIO; a glycogen synthase kinase-3β inhibitor, to activate the Wnt-signaling pathway) (Calbiochem), and cultured in 96-well Corning Costar Ultra-Low attachment multiwell plates (Sigma-Aldrich) for 3 days. On day 3, an additional 100 µL DM without BIO was added to each well. On day 5, individual embryoid bodies (EBs) were transferred to 100-mm gelatin-coated dishes (250 EBs per dish). On days 6, 7, 10, 11, 14, and 15 the medium was exchanged for serum-free Modified Eagle's Medium (MEM; Invitrogen) with insulin transferrin-selenium-X (Invitrogen). On days 8, 9, 12, and 13, the medium was exchanged for Glucose-free DMEM (no glucose, no pyruvate, Invitrogen) supplemented with 4 mmol/L lactic acid (Wako Pure Chemical) for purification of cardiomyocytes. On day 16, the contracting cell clusters were used as cardiomyogenically differentiated iPSCs (959A2-1 CMs, 959C1-1 CMs, 956F-1 CMs: iPSC-CMs). The protocol and purification process are illustrated in Figure 1a.

Adult cardiac tissue from B6 mice (CLEA) was used as a control. Male B6 mice (8 weeks old) were sacrificed by intravenous administration of potassium chloride under inhalation anesthesia of isoflurane, and heart tissue from the left ventricle was harvested for further studies and labeled “Heart”.

### Immunohistochemistry analysis

IPSC-CMs were dissociated with 0.25% trypsin-EDTA and then fixed with 4% paraformaldehyde. The cells were stained with the following primary antibodies: mouse anti-α-actinin antibody (Sigma-Aldrich) and rabbit anti-troponin I antibody (Abcam), and then visualized by the following secondary antibodies: Alexa Fluor 488 donkey anti-mouse IgG (Invitrogen) and Alexa Fluor 594 goat anti-rabbit IgG (Invitrogen). The nucleus of the cells were stained with 4′, 6-Diamidino-2-phenylindole dihydrochloride (DAPI) and then observed with a confocal laser scanning microscopy FV1200 (Olympus).

### Ca^2+^ transient measurement and pharmacological analysis

5 µM Fluo-8 regents (AAT Bioquest, Inc.) in serum-free MEM was added to iPSC-CMs after the cells were washed with phosphate buffered saline. The cells were incubated at 37°C for 30 min and then observed with a fluorescence microscopy. Fluorescence intensity of Fluo-8 dye was sequentially measured using iQ2 software (ANDOR) pre and post the administration of 1 µM isoproterenol.

### Flow cytometry

IPSC-CMs were dissociated with 0.25% trypsin-EDTA and then fixed with CytoFix fixation buffer (BD) for 20 min. The cells were permeabilized with Perm/Wash buffer (BD) at room temperature for 10 min and then incubated with mouse anti-troponin T antibody (Thermo) for 30 min. Cells were washed with Perm/Wash buffer prior to incubation with the Alexa Fluor 488 rabbit anti-mouse IgG secondary antibody (Invitrogen) at room temperature for 30 min. These cells were analyzed on a FACS Canto II (BD).

### Characterization of *N*-glycans derived from iPSCs, iPSC-CM, and Heart

All experimental procedures, including chromatography conditions and glycosidase treatments, have been described previously [23]. Cultured undifferentiated iPSCs, iPSC-CMs, and the heart tissue were treated with chloroform–methanol, then subjected to proteolysis with chymotrypsin and trypsin, followed by glycoamidase A digestion to release *N*-glycans. After removal of peptides, the reducing ends of the *N*-glycans were derivatized with 2-aminopyridine (Wako). This mixture was applied to a diethylaminoethyl (DEAE) column (Tosoh) or a TSK-gel Amide-80 column (Tosoh); each fraction from the amide column was then applied to a Shim-pack HRC-octadecyl silane (ODS) column (Shimadzu). The elution times of individual peaks from the amide-silica and ODS columns were normalized to a pyridylamino (PA)-derivatized isomalto-oligosaccharide with a known degree of polymerization, and are represented as glucose units (GU). Thus, each compound from these two columns provided a unique set of GU values, which corresponded to the coordinates of the 2D HPLC map. The PA-oligosaccharides were identified by comparison to the coordinates of ∼500 reference PA-oligosaccharides in a homemade web application, GALAXY (http://www.glycoanalysis.info/galaxy2/ENG/index.jsp) [24]. The calculated HPLC map based on the unit contribution values was used to estimate some high-mannose type PA-oligosaccharides. The PA-oligosaccharides were co-chromatographed with the reference to PA-oligosaccharides on the columns to confirm their identities. PA-glycans that did not correspond to any of the *N*-glycans registered in GALAXY were trimmed by exoglycosidase to produce a series of known glycans [25].

### Mass spectrometry

PA-oligosaccharides were analyzed by matrix-assisted laser desorption/ionization time-of-flight mass spectrometric (MALDI-TOF/MS). The matrix solution was prepared as follows: 10 mg of 2,5-Dihydroxybenzoic acid (Sigma) was dissolved in 1∶1 (v/v) acetonitrile/water (1 mL). Stock solutions of PA-glycans were prepared by dissolving them in pure water. One microliter of a sample solution was mixed on the target spot of a plate with 1 µL matrix solution and then allowed to air-dry. MALDI-TOF/MS data were acquired in the positive mode on an AXIMA-CFR (Shimadzu) operated in linear mode.

### Materials

Glycoamidase A from sweet almond, α-mannosidase, β-galactosidase, and β-*N*-acetylhexosaminidase from jack bean were purchased from Seikagaku Kogyo (Tokyo, Japan). α-Galactosidase from coffee bean was purchased from Oxford GlycoSciences (Oxford, UK). Trypsin and chymotrypsin were obtained from Sigma (St. Louis, MO). Pronase protease from *Streptomyces griseus* was from Calbiochem (San Diego, CA). The pyridylamino (PA) derivatives of isomalto-oligosaccharides 4–20 (indicating the degree of polymerization of glucose residues) and reference PA-oligosaccharides were purchased from Seikagaku Kogyo.

### Semi-quantitative PCR

DNA-free total RNA was extracted with the RNeasy RNA isolation Kit (Qiagen) and reverse-transcribed into cDNA using Omniscript reverse transcriptase (Qiagen), then analyzed by quantitative real-time PCR on an ABI PRISM 7700 thermocycler (Applied Biosystems) with the following TaqMan gene expression assays (Applied Biosystems): ST3Gal-III (Gal β1-3(4) GlcNAc α-2, 3-sialyltransferase), Mm00493353_m1; ST4Gal-IV (Gal β1-4(3) GlcNAc α-2, 3-sialyltransferase), Mm00501503_m1; ST6Gal-I (Gal β1-4 GlcNAc α-2, 6-sialyltransferase), Mm00486119_m1; CMAH (cytidine monophosphate-*N*-acetylneuraminic acid hydroxylase), Mm00483341_m1; GAPDH (glyceraldehyde-3-phosphate dehydrogenase), and Mm03302249_g1 and with SYBR Green dye (Applied Biosystems) using the following primers: Nkx2.5 F, 5′- CAAGTGCTCTCCTGCTTTCC -3′ R, 5′- GGCTTTGTCCAGCTCCACT -3′; αMHC (α-myosin heavy chain) F, 5′- GAGATTTCTCCAACCCAG -3′ R, 5′- TCTGACTTTCGGAGGTACT-3′; ANP (atrial natriuretic peptide) F, 5′- AAAGAAACCAGAGTGGGCAGAG -3′ R, 5′- CCAGGGTGATGGAGAAGGAG -3′; Isl1 F, 5′- TTTCCCTGTGTGTTGGTTGC -3′ R, 5′- TGATTACACTCCGCACATTTCA -3′; GAPDH F, 5′- CCAGTATGACTCCACTCACG -3′ R, 5′- GACTCCACGACATACTCAGC -3′. All experiments were performed by the relative standard curve method in three independent, triplicate experiments. Statistical comparison of the data was performed by Student's t-test.

## Results

### Highly purified cardiomyocytes derived from iPSCs

**Figure 2:**
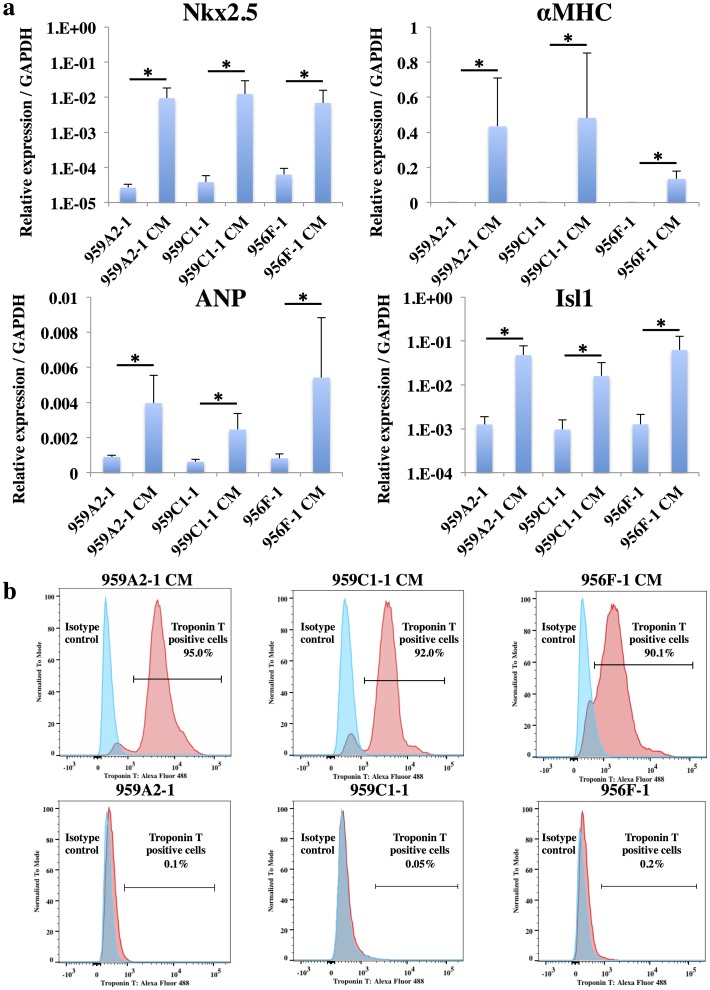
Highly purified iPSC-CMs expressing cardiomyocyte marker genes. (a) Transcript expression of Nkx2.5, αMHC, ANP and Isl1 in the iPSCs and the iPSC-CMs were analyzed by real-time PCR. Results are expressed as the mean ± standard deviation. *P<0.05. (b) IPSC-CMs and iPSCs stained with anti-troponin T antibody or the isotype control, followed by Alexa Fluor 488-conjugated anti-mouse IgG antibody, were analyzed by flow cytometry.

**Figure 3:**
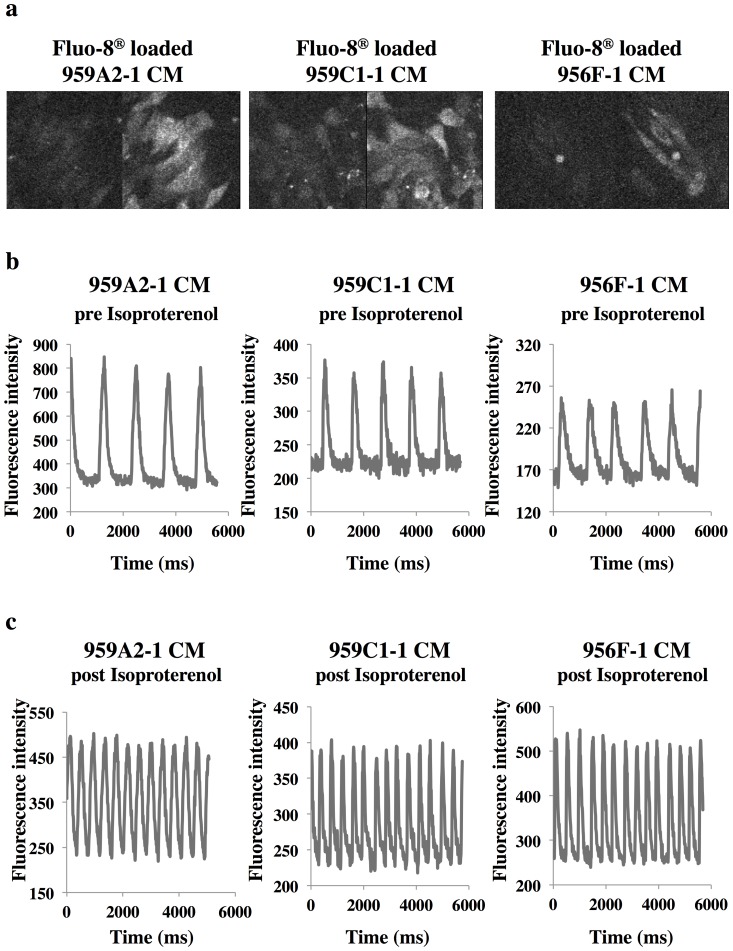
Ca^2+^ transient measurement of iPSC-CMs pre and post the administration of isoproterenol. (a) Fluo-8 loaded iPSC-CMs at the time of low (left) and high (right) fluorescence. (b), (c) Sequentially measured fluorescence intensity of Fluo-8 loaded iPSC-CMs pre (b) and post (c) the administration of 1 µM isoproterenol.

Cardiomyogenic differentiation was induced in murine iPSCs by using a slightly modified culture protocol (Figure 1a). The iPSC-CMs showed significantly higher expressions of Nkx2.5, αMHC, ANP and Isl1 than undifferentiated iPSCs by semi-quantitative real-time PCR (Figure 2a), and showed sarcomere structures observed by immunohistological staining of α-actinin and troponin I (Figure 1b). The iPSC-CMs were functional with Ca^2+^ transient measurement (Figure 3a, b) and their beating rates were increased by the administration of isoproterenol (Figure 3c), meaning they had β-adrenergic receptors. Nearly all of the iPSC-CMs exhibited spontaneous and regular beating at room temperature (Video S1). The differentiation efficiency of murine iPSC was evaluated by flow cytometry analysis. More than 95% of the 959A2-1 CMs, 92% of the 959C1-1 CMs and 90% of the 956F-1 CMs were positive for troponin T (Figure 2b), while the undifferentiated iPSCs rarely expressed troponin T (Figure 2b).

### *N*-Glycans isolated from iPSCs, iPSC-CM, and Heart

**Figure 4:**
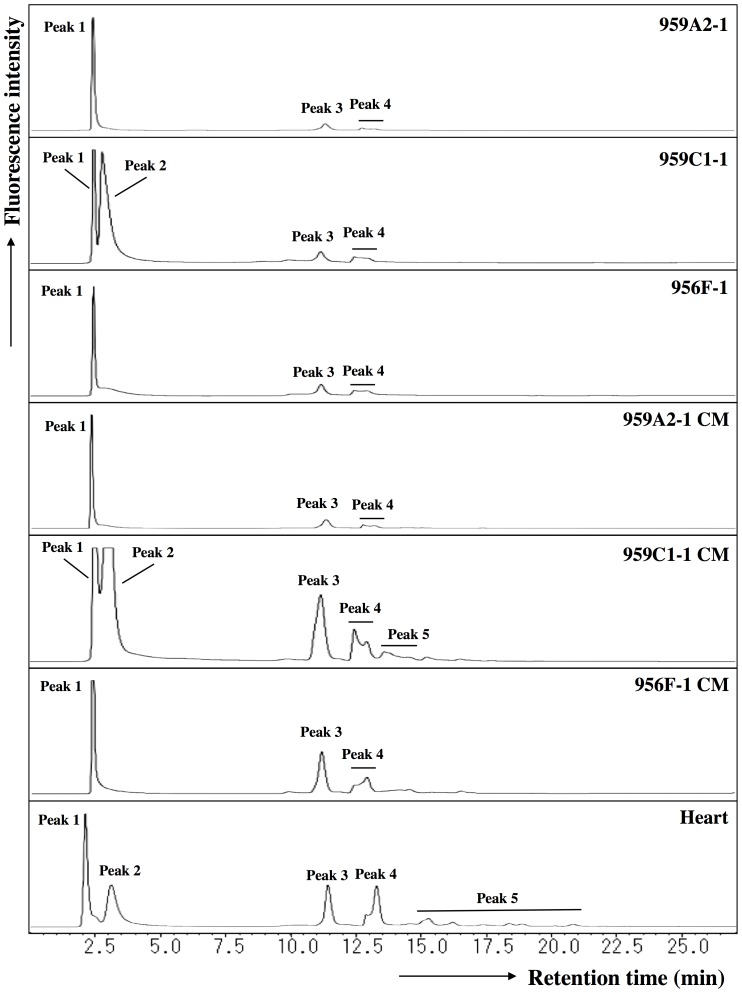
Anion-exchange DEAE elution profiles of PA-glycans. PA-glycans were fractionated according to their sialic acid content as neutral (peak 1), monosialyl (peak 3), and disialyl (peak 4) oligosaccharide fractions. Peaks 2 and 5 represent fractions containing no detectable PA-oligosaccharides.

*N*-glycans extracted from undifferentiated iPSCs (959A2-1: 26 mg, 959C1-1: 11 mg and 956F-1: 10 mg of protein), iPSC-CM (959A2-1 CM: 15 mg, 959C1-1 CM: 12 mg and 956F-1 CM: 5.5 mg of protein), and the B6 heart muscle (82 mg of protein) were separated on a diethylaminoethyl (DEAE) column into five peaks, based on increasing acidity. Peak 1 represented a neutral (N) fraction, peak 3 a monosialyl (M) fraction, and peak 4 a disialyl (D) fraction. Glycan fractions in each of these peaks were as follows: iPSCs yielded 97% N, 0.5% M, 2.6% D (959A2-1), 98% N, 0.7% M, 1.1% D (959C1-1) and 96% N, 1.1% M, 3.1% D (956F-1) peak areas, iPSC-CMs yielded 89% N, 6.4% M, 4.4% D (959A2-1 CM), 79% N, 16% M, 4.8% D (959C1-1 CM) and 82% N, 10% M, 7.9% D (956F-1 CM) and Heart yielded 55% N, 19% M, and 25% D (Figure 4).

**Figure 5:**
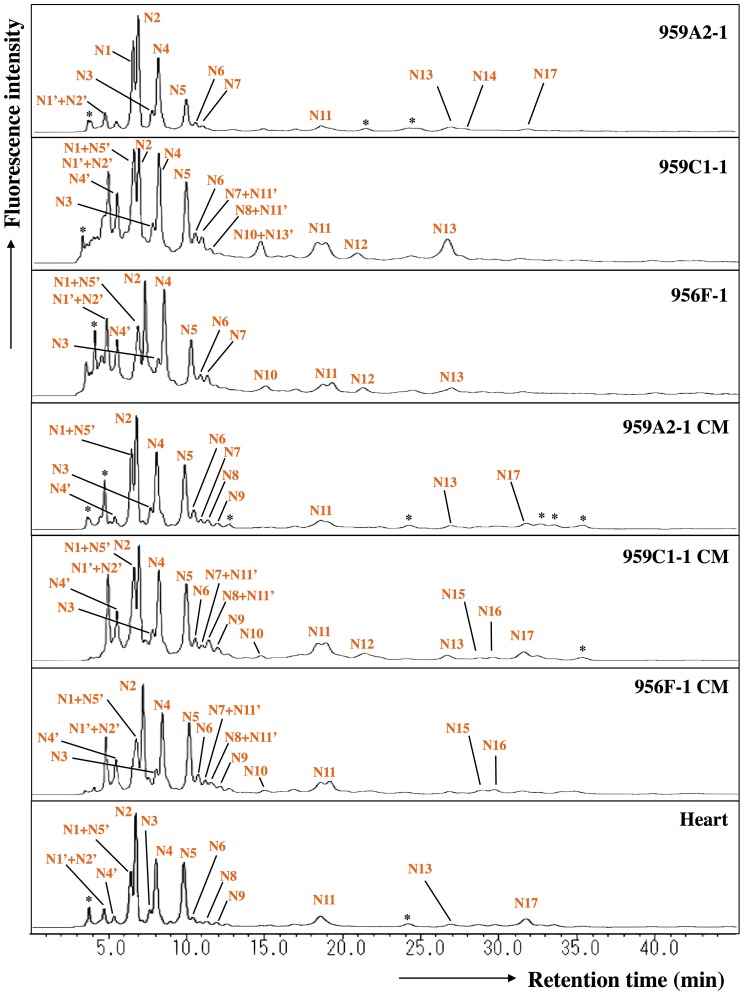
Reverse-phase ODS elution profiles of the neutral PA-glycans. The neutral fractions were individually applied to the ODS column and eluted according to their hydrophobicity. N1′, N2′, N4′, N5′ and N11′: epimerization of N1, N2, N4, N5 and N11. *Fractions containing no detectable PA-oligosaccharides.

**Figure 6:**
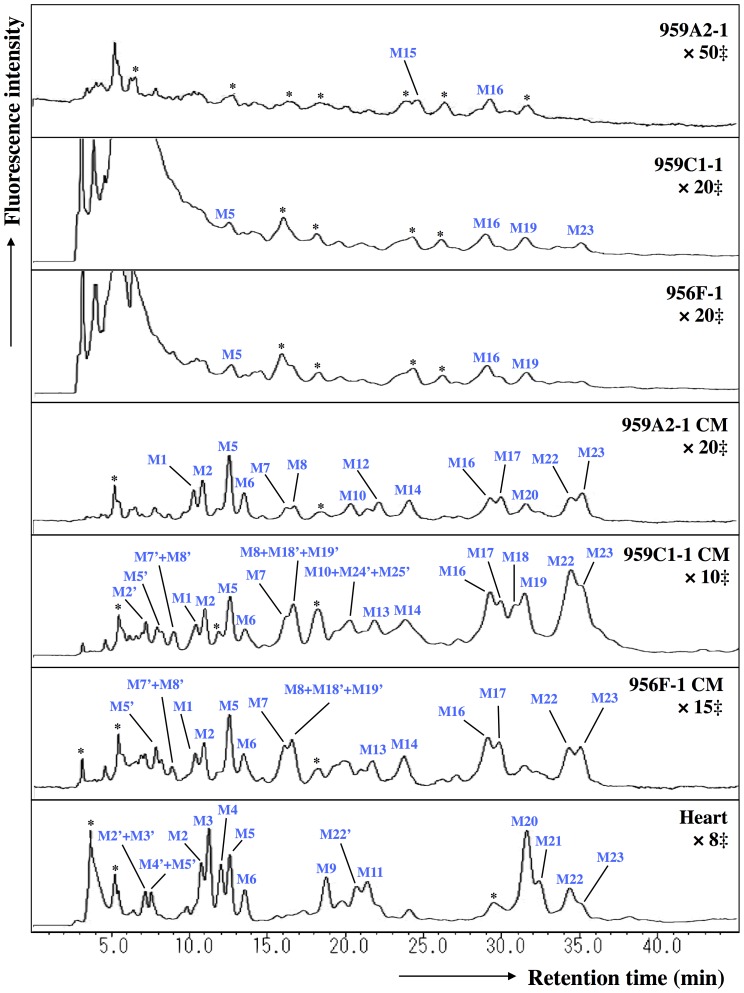
Reverse-phase ODS elution profiles of monosialyl PA-glycans. The monosialyl fractions were individually applied to the ODS column and eluted according to their hydrophobicity. M2′, M3′, M4′, M5′, M7′, M8′, M18′, M19′, M22′, M24′ and M25′: epimerization of M2, M3, M4, M5, M7, M8, M18, M19, M22, M24 and M25. *Fractions containing no detectable PA-oligosaccharides. ‡Magnification ratio to the fluorescence intensity of asialoglycan of each sample.

**Figure 7:**
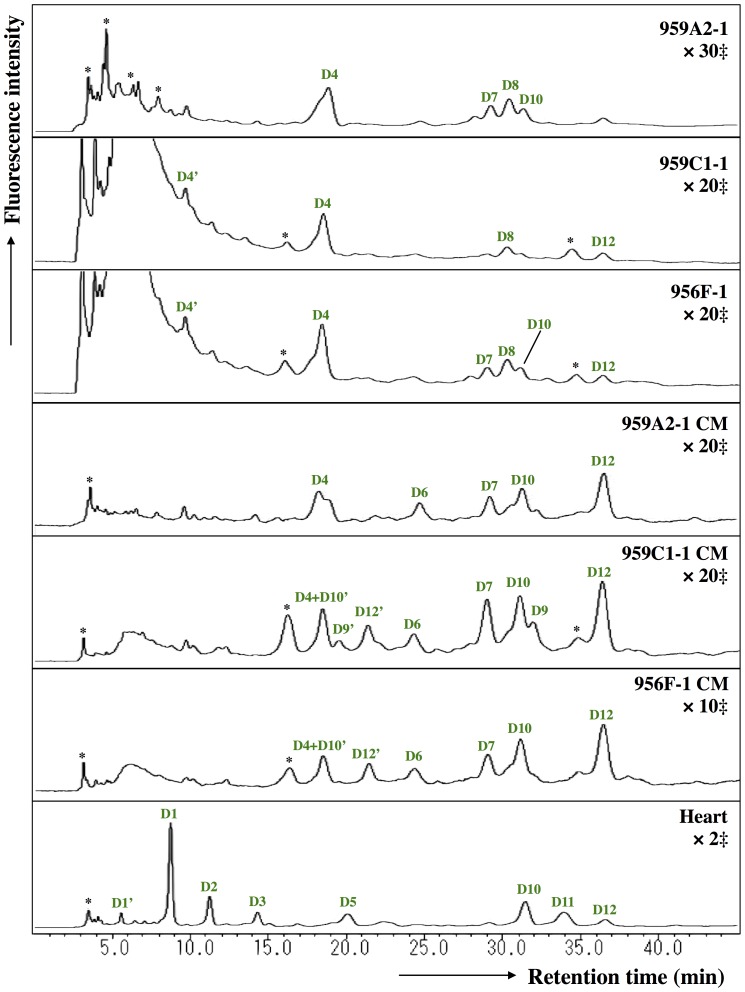
Reverse-phase ODS elution profiles of disialyl PA-glycans. The disialyl fractions were individually applied to the ODS column and eluted according to their hydrophobicity. D1′, D4′, D10′ and D12′: epimerization of D1, D4, D10 and D12; *Fractions containing no detectable PA-oligosaccharides. ‡Magnification ratio to the fluorescence intensity of asialoglycan of each sample.

The ODS column separated the neutral fraction (Peak 1) into fractions N1–N17 (Figure 5), the monosialyl fraction (Peak 3) into fractions M1–M23 (Figure 6), and the disialyl fraction (Peak 4) into fractions D1–D12 (Figure 7). The signatures of each fraction differed between groups. These ODS fractions were individually fractionated on an amide column and analyzed by MALDI-TOF/MS. The N2, M6, M11, M14, M20, D4, D5, and D10 fractions contained two types of *N*-glycans, and the N6, N9, N11 and M2 fractions three types (data not shown). Thus, 68 different *N*-glycans were isolated from iPSCs, iPSC-CMs, and Heart.

### Structures of *N*-Glycans isolated from iPSCs, iPSC-CM, and Heart

The isolated *N*-glycans were analyzed by means of a mapping technique based on their HPLC elution positions and MALDI-TOF/MS data. The coordinates of 54 *N*-glycans coincided with those for known references in the GALAXY database and their structures were identified. The coordinates for N9-3, M8, M11-2, M12, M13, M15, M17, M18, M19, M20-2, M21, M23, D8 and D9 did not correspond to known references.

**Figure 8:**
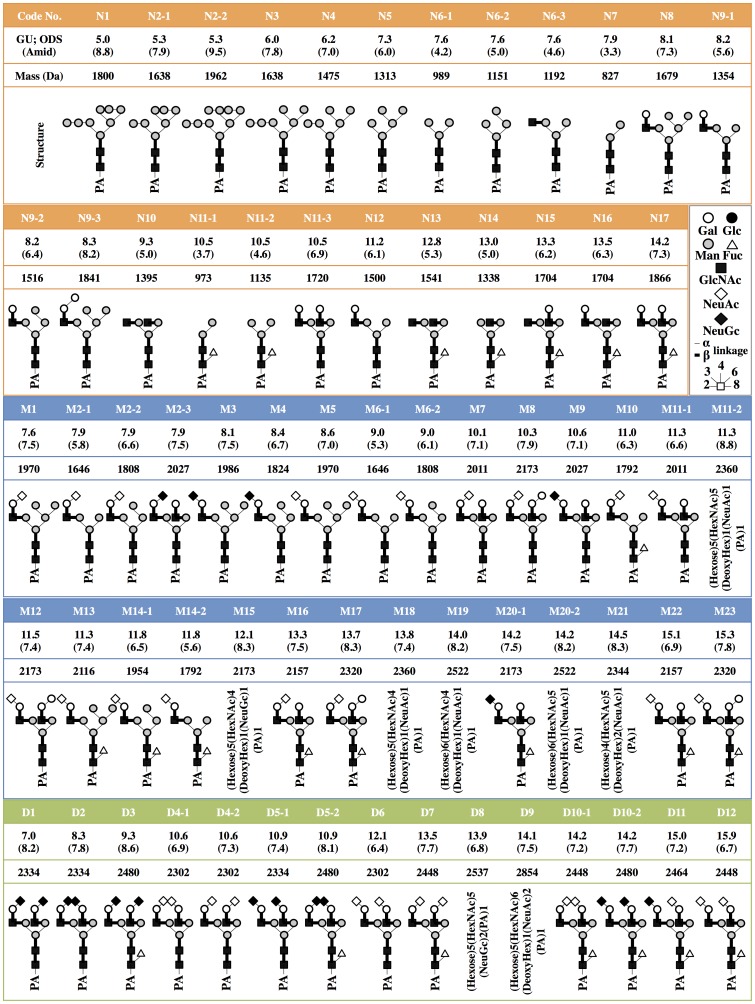
Structures of neutral, monosialyl, and disialyl PA-oligosaccharides in iPSCs, iPSC-CM, and heart cells. Glucose units (GU) were calculated from the peak elution times for the ODS column in Figure 5, 6 and 7, and the amide column (data not shown). Average mass (Mass) calculated from the *mlz* values of [M+Na]^+^ or [M+H]^+^ ion for neutral, [M-H]^−^ ion for monosialyl, and [M-H]^−^ & [M+Na-2H]^−^ ions for disialyl PA-oligosaccharides.

*N*-glycans N9-2, M8, M12, M17, and M23 were trimmed by α-galactosidase but not by β-galactosidase or *N*-acetylglucosamidase. Their structures fit GALAXY references H5.12, 1A1-200.4, 1A3-200.4, 1A1-210.4, and 1A3-210.4, respectively. The galactosyl structures were then identified as Galα1-6Gal, because of the α-galactosidase-driven MS shifts. The structure of the M13 was identified by the coincidence with a GALAXY reference 1A2-H5.12 after being trimmed by α-L-fucosidase. The other *N*-glycans M11-2, M15, M18, M19, M20-2, M21, D8 and D9 were not identified in this study because they did not correspond to GALAXY references even after α-galactosidase digestions. They are described in Figure 8 and Table S1-S5 with their proposed formulas based on MALDI-TOF/MS data.

### High-mannose *N*-Glycans were reduced by cardiomyogenic differentiation

**Figure 9:**
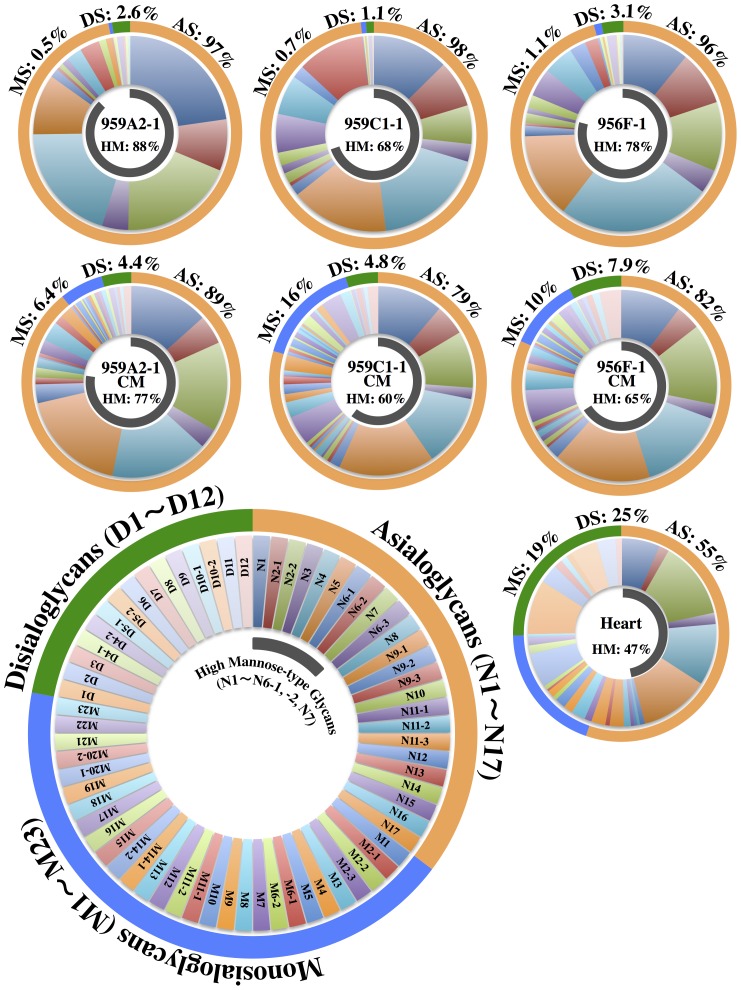
Relative quantities of neutral, monosialyl, and disialyl PA-oligosaccharides in iPSCs, iPSC-CM, and heart cells. Relative quantities of each glycan, calculated from the peak area in Figure 5, 6 and 7 vs. total *N*-glycan content in each cell, were expressed in the doughnut charts. Relative quantities of the asialoglycans, the monosialoglycans and the disialoglycans were showed outside of the charts, and relative quantities of the high mannose type glycans were showed inside of the charts. Asialoglycan (AS): the total volume of N1-N17; Monosialoglycan (MS): the total volume of M1-M23; Disialoglycan (DS): the total volume of D1-D12, High mannose-type glycan (HM): the total volume of N1–N6-1, N6-2, N7.

The quantity of high-mannose *N*-glycans (HM) calculated from the total volume of N1–N6-2, N7 was highest in the iPSCs (959A2-1: 87.7%, 959C1-1: 68.3% and 956F-1: 78.2%), lower in the iPSC-CMs (959A2-1 CM: 77.4%, 959C1-1 CM: 60.0% and 956F-1 CM: 65.1%), and lowest in the Heart (46.9%). The quantity of monofucosylated, difucosylated, and other types of *N*-glycans were greater in the iPSC-CMs and Heart (Figure 8, 9).

### Sialyl *N*-glycans increased with cardiomyogenic differentiation

**Figure 10:**
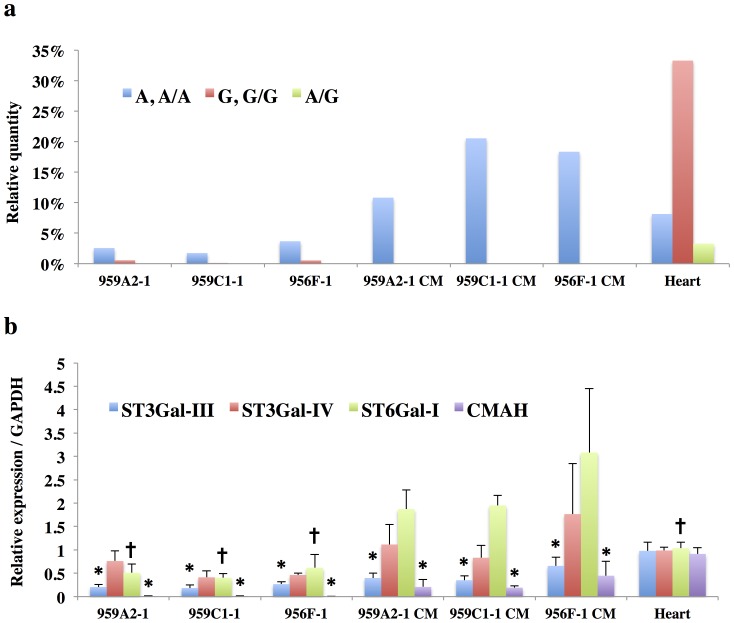
Rarely expressed NeuGc-containing glycans in iPSCs and iPSC-CMs. (a) Relative quantities of NeuAc- and NeuGc-containing glycans; Monosialoglycans containing NeuAc and Disialoglycans containing two NeuAc (A, A/A): the total volume of M1, M2-1, M2-2, M5-M8, M10-M14, M16-M19, M20-2, M21-M23, D4-1, D4-2, D6, D7, D9, D10-1, D12, Disialoglycan containing NeuAc and NeuGc (A/G): D11, Monosialoglycan containing NeuGc and Disialoglycan containing two NeuGc (G, G/G): the total volume of M2-3, M3, M4, M9, M15, M20-1, D1-D3, D5-1, D5-2, D8, D10-2. (b) Transcript expression of ST3Gal-III, ST3Gal-IV, ST6Gal-I, and CMAH; Transcript expression of glycosyltransferases in iPSCs, iPSC-CM, and heart cells was analyzed by real-time PCR. Results are expressed as the mean ± standard deviation. *P<0.05 vs. Heart, †P<0.05 vs. iPSC-CM (all of the 959A2-1 CM, 959C1-1 CM and 956F-1 CM).

The quantity of monosialyl *N*-glycans (MS) calculated from the total volume of M1–M23 increased in iPSC-CMs (959A2-1 CM: 6.4%, 959C1-1 CM: 15.7% and 956F-1 CM: 10.5%) and Heart (19%) and were low in iPSCs (959A2-1: 0.5%, 959C1-1: 0.7% and 956F-1: 1.1%). The disialyl *N*-glycans (DS; D1–D12) yielded a similar pattern. The quantity of asialyl *N*-glycans (AS; N1–N17) decreased in iPSC-CMs (959A2-1 CM: 89.2%, 959C1-1 CM: 79.4% and 956F-1 CM: 81.7%) and Heart (55.3%) in comparison to the iPSCs (959A2-1: 96.9%, 959C1-1: 98.1% and 956F-1: 95.8%) (Figure 9, 10).

### Rarely expressed *N*-glycans

The sialic acids identified in this study were either *N*-acetyl neuraminic acid (NeuAc) or *N*-glycolyl neuraminic acid (NeuGc). The quantity of monosialyl and disialyl *N*-glycans containing only NeuAc (A, A/A) was lowest in iPSCs (959A2-1: 2.5%, 959C1-1: 1.7% and 956F-1: 3.7%) and similar in iPSC-CMs (959A2-1 CM: 10.6%, 959C1-1 CM: 21% and 956F-1 CM: 18%) and the Heart (8%). The quantity of monosialyl and disialyl *N*-glycans containing only NeuGc (G, G/G) was markedly higher in the Heart (32.8%) than in iPSCs (959A2-1: 0.6%, 959C1-1: 0.1% and 956F-1: 0.5%) or iPSC-CMs (959A2-1 CM: 0%, 959C1-1 CM: 0% and 956F-1 CM: 0%) (Figure 10a).

Expression of glycosyl transferase, ST3Gal-III, ST3Gal-IV, ST6Gal-I, and CMAH in the iPSCs, iPSC-CMs, and Heart was assessed by RT-PCR to explore the glycan structures responsible for the differences between groups. The Heart expressed high levels of CMAH (0.91±0.13/GAPDH); levels in the iPSCs and iPSC-CMs were markedly lower (iPSCs: 959A2-1 0.011±0.0065/GAPDH, 959C1-1 0.013±0.0070/GAPDH, 956F-1 0.0045±0.0042/GAPDH, P<0.05; iPSC-CM: 959A2-1 CM 0.21±0.16/GAPDH, 959C1-1 CM 0.19±0.04, 956F-1 CM 0.45±0.31, P<0.05). Expression of ST3Gal-III was significantly higher in the Heart (0.98±0.13/GAPDH) than in iPSCs (959A2-1: 0.21±0.05/GAPDH, 959C1-1: 0.18±0.07/GAPDH, 956F-1: 0.27±0.05/GAPDH) and iPSC-CMs (959A2-1 CM: 0.40±0.10/GAPDH, 959C1-1 CM: 0.35±0.09/GAPDH, 956F-1 CM: 0.66±0.18); expression of ST3Gal-IV did not differ between groups. ST6Gal-I expression was significantly higher in iPSC-CMs (959A2-1 CM: 1.87±0.41/GAPDH, 959C1-1 CM: 1.95±0.22/GAPDH, 956F-1 CM: 3.08±1.27/GAPDH) than in iPSCs (959A2-1: 0.51±0.18/GAPDH, 959C1-1: 0.40±0.09/GAPDH, 956F-1: 0.62±0.29/GAPDH) and the Heart (1.04±0.13/GAPDH) (Figure 10b).

## Discussion

Sixty-eight different *N*-glycans were isolated from iPSCs, iPSC-CMs, and the Heart. The structures of 60 *N*-glycans were identified, based on their HPLC elution peaks (Figure 8, Table S1-S5). Each preparation contained a combination of neutral, monosialyl, and disialyl *N*-glycans.

The molar ratios of high-mannose, monofucosylated, and difucosylated *N*-glycans were substantially different between groups (Figure 9), although no clear differences in the abundance of these glycans were found. The decrease in high-mannose *N*-glycans and increase of fucosylated *N*-glycans in iPSC-CMs versus iPSCs is consistent with a previous report on a comparison of ESC derived cardiomyocytes to undifferentiated ESCs [18]. Generally, all *N*-glycans are synthesized from the high-mannose type by a large array of sequentially and competitively acting biosynthetic enzymes located throughout the endoplasmic reticulum and Golgi apparatus [26], indicating that the high-mannose type of *N*-glycans could be categorized as a marker of immaturity. In this study, the high-mannose *N*-glycans were highest in the immature iPSC and lowest in the Heart, or mature tissue; thus, the quantity of high-mannose-type *N*-glycans might be an indicator of maturity in iPSC-derivatives and the iPSC-CMs in our protocol may still be immature in comparison to cardiac tissue.

Clear differences in glycan abundance were observed, such as hybrid and complex types represented by N9-1, N9-3, N15, N16, M1, M2-1, M2-2, M7, M8, M10, M12, M13, M14-1, M14-2, M17, M18, M20-2, D6 and D9 in iPSC-CMs, M2-3, M3, M4, M9, M11-1, M11-2, M20-1, M21, D1, D2, D3, D5-1, D5-2, D10-2 and D11 in Heart and N14 and M15 in iPSCs; these may also be indicators of maturation stage. In addition, expression of monosialyl and disialyl *N*-glycans in iPSC-CMs fell between the levels observed in the iPSCs and Heart, as were the molar ratios, indicating that the iPSC-CMs may still be immature stage. While many *N*-glycolyl neuraminic acid (NeuGc) structures were detected in the Heart, iPSCs and iPSC-CMs did not contain NeuGc in their sialyl structures, except for D8. Moreover, the molar ratio of NeuAc was low in iPSCs and iPSC-CMs. This finding is one of the clearest differences between iPSCs or iPSC-CMs and Heart cells.

The proposed spectra-based composition of the D8 glycans in iPSCs was [(Hexose)5(HexNAc)5(NeuGc)2(PA)1], indicating that it contains NeuGc. However, D8 might be quite a rare exception because transcript levels of CMAH, which catalyzes the conversion of NeuAc to NeuGc, was quite low in iPSCs in comparison to the Heart. This data suggests that during the process of reprograming, iPSCs suppress or eliminate CMAH activity. We conclude that iPSCs contain less sialic acid (especially NeuGc) and high-mannose structures are abundant in the *N*-glycans. In contrast, heart cells produce numerous sialyl-*N*-glycans, especially NeuGc. Transcript levels of CMAH tended to increase in iPSC-CMs relative to iPSCs, suggesting cardiomyogenic differentiation may induce expression of CMAH. If the iPSC-CMs could be matured more closely to the Heart by some additional methods of culture, the quantity of high mannose type of *N*-glycans might decrease more closely to the Heart, and might produce *N*-glycans containing NeuGc, followed by the expression of CMAH.

A terminal NeuGc, the Hanganutziu-Deicher (H-D) epitope [27], is widely distributed in the animal kingdom with the exception of humans and chickens. Expression of NeuGc is controlled by CMAH activity. Irie et al. [28] and Chou et al. [29] cloned the cDNA for human CMAH and reported that the *N*-terminal truncation of human CMAH is caused by deletion of Exon 6, a 92-base pair segment in the genomic DNA. Expression of this truncation in the heart eliminates NeuGc in sialyl structures. If human iPSCs or iPSC-CMs do not express CMAH in the same way as murine iPSCs or iPSC-CMs, there may be no difference between human iPSCs, iPSC-CMs, and the human Heart. Further study on human iPSC-CM will be needed to completely understand the features of the sialyl acid of *N*-glycans.

It was reported that human iPSCs produced α2,6sialyl glycans but did not contain α2,3sialyl structures, in contrast to human fibroblast, the origin of iPSCs, which produced α2,3sialyl but not α2,6sialyl structures [30], [31]. The murine iPSCs in this study contained α2,3sialyl structures in NeuAc, M5, M23, D4-1, D10-1 and D12, and the iPSC-CMs produced α2,3 and α2,6sialyl structures in NeuAc. These differences may be due to variations between species, because mouse Heart cells also contained α2,3 and α2,6sialyl structures in NeuGc. Further studies are needed to characterize the glycome shift in the production and differentiation of iPSCs.

Type I Lactose structures were not detected, although over 98% of glycans in each cell were accounted for in this study. The *N*-glycans of N9-3, M8, M12, M17, and M23, which were identified after α-galactosidase digestion, contained Galα1-6Gal, not only in the neutral glycans but also in the monosialyl *N*-glycans of the iPSC-CM preparation. The same structure was not found in iPSCs, but only one structure, M23, was present in Heart cells. Therefore, in iPSC-CMs, Galα1-6Gal enzyme activity appears to be up-regulated in comparison to wild-type myocardium, although enzyme activity was not assessed by RT-PCR because of the limited availability of genetic sequence data.

The D8 was identified in all of three iPSC lines and not in the iPSC-CMs and Heart. This structure, unfortunately not identified in this study, may be useful as markers of undifferentiated iPSCs in the same way as well-known pluripotency biomarkers such as stage-specific embryonic antigens (SSEA)-3, SSEA-4 (glycosphingolipids) [32].

Previous MALDI-TOF/MS and MS/MS studies concluded that many kinds of *N*-glycans are found in organs and cells. The number of detected *N*-glycans is attributed to the sensitivity of the MS and HPLC methods employed. That is, MS data are sensitive and can be rapidly obtained, but a glycan structure is identified based only on the calculated molecular weight. Therefore, discriminating between isomeric structures is difficult. On the other hand, it thus appears that the accuracy of the data presented here using HPLC mapping in conjunction with a MALDI-TOF technique provides much more detailed information. Our data were used to identify the representative features of each *N*-glycan in these three cell types.

There may be a concern that the heart tissue used in this study contains connective tissues, vessels or nerves other than cardiomyocytes. Therefore, some of the *N*-glycans detected from the Heart sample might be derived from the tissues other than cardiomyocytes. However, heart is majority composed by cardiomyocytes, and furthermore, even if a small amount of *N*-glycans derived from connective tissues were contaminated in the Heart sample, the main evidences in this study, such as the proportion of the high-mannose type *N*-glycans, the ratio of the active sialyltransferase genes, the existence of NeuGc, and the uncommonness of Galα1-6 Gal, are essentially not affected.

In summary, murine iPSCs were rich in high-mannose type *N*-glycans but very poor in sialyl type *N*-glycans. Murine heart tissue contained a relatively low volume of high-mannose glycans, but was very rich in neuraminic acid, especially NeuGc type sialyl structures. Under these conditions, the volume of each type of glycan was similar for iPSC-CMs and iPSCs. That is, they were rich in high-mannose and relatively poor in sialyl type *N*-glycans by volume. In addition, most of the sialyl structures of the iPSC-CMs were different from those of the Heart, and the iPSC-CMs expressed no NeuGc. Moreover, the iPSC-CMs produced several unique glycans with the Galα1-6Gal structure. These results provide important data that can be useful in future clinical iPSC studies.

It is quite important to investigate the meaning of *N*-glycans transitions during the cardiomyogenic differentiation presented in this study, for deeply understanding the relationship between the *N*-glycan expression and cardiomyogenic differentiation. Knock-out or knock-down of the genes related to cardiomyogenic differentiation or glycosylation may be useful for such purpose. However, the *N*-glycan signature in the cell surface is determined by a variety of the genes. Knock-out or knock-down of a single gene related to cardiomyogenic differentiation would alter an array of gene expressions, such as sarcomere proteins, transcriptional factors, or cell surface proteins, all of which would affect the signature of *N*-glycans in the cell surface. Therefore, the data interpretation for relationship between expression of a single gene and *N*-glycan signature would be difficult. Some different experimental approach may be needed to investigate the meaning of change in *N*-glycan expression during cardiomyogenic differentiation.

## Supporting Information

Table S1 **Structures and relative quantities of neutral (Table S1, S2) PA-oligosaccharides derived from iPSC, iPSC-CM, and heart cells.** a. Glucose units (GU) were calculated from the peak elution times of the peaks obtained from the ODS column in Figure 5, 6, 7 and the Amide column (data not shown). b. Average mass calculated from the *mlz* values of [M+Na]^+^ or [M+H]^+^ ion for neutral, [M-H]^−^ ion for mono-sialyl, and [M-H]^−^ & [M+Na-2H]^−^ ions for di-sialyl PA-oligosaccharides. c. PA-oligosaccharide structures. d. mol% was calculated from the peak area versus total *N*-glycan content in each cell
(TIFF)

Table S2 **Structures and relative quantities of neutral (Table S1, S2) PA-oligosaccharides derived from iPSC, iPSC-CM, and heart cells.**
(TIFF)

Table S3 **Structures and relative quantities of monosialyl (Table S3, S4) PA-oligosaccharides derived from iPSC, iPSC-CM, and heart cells.**
(TIFF)

Table S4 **Structures and relative quantities of monosialyl (Table S3, S4) PA-oligosaccharides derived from iPSC, iPSC-CM, and heart cells.**
(TIFF)

Table S5 **Structures and relative quantities of disialyl PA-oligosaccharides derived from iPSC, iPSC-CM, and heart cells.**
(TIFF)

Video S1 (MP4)
